# Trends of routine childhood vaccination status in Afghanistan over the last two decades (1999–2023)

**DOI:** 10.1186/s41182-025-00830-5

**Published:** 2025-11-02

**Authors:** Ghulam Raza Mohammadyan, Seyed Aria Nejadghaderi, Hamid Sharifi, Mohammad Mehdi Gouya, Seyed Mohsen Zahraei, AliAkbar Haghdoost

**Affiliations:** 1 HIV/STI Surveillance Research Center, and WHO Collaborating Center for HIV Surveillance, Institute for Futures Studies in Health, Kerman University of Medical Sciences, Kerman, Iran; 2 Knowledge Hub for Migrant and Refugee Health, Institute for Futures Studies in Health, Kerman University of Medical Sciences, Kerman, Iran; 3 Institute for Global Health Sciences, University of California, San Francisco, CA USA; 4 Faculty of Public Health, Iran University of Medical Sciences, Tehran, Iran; 5 Center for Communicable Disease Control, Ministry of Health and Medical Education, Tehran, Iran; 6 Social Determinants of Health Research Center, Institute for Futures Studies in Health, Kerman University of Medical Sciences, Kerman, Iran

**Keywords:** Diphtheria–Tetanus–Pertussis vaccine, Vaccination coverage, BCG vaccine, Hepatitis B vaccines, Measles vaccine, Afghanistan, Immunization

## Abstract

**Background:**

Global vaccine coverage improved substantially. In Afghanistan, routine immunization has been expanding since 1978 but remains inadequate, contributing to consistently high under-five mortality rates. This time-trend analysis focused on national routine childhood immunization coverage and the number of Expanded Programme on Immunization (EPI) centers in Afghanistan from 1999 to 2023.

**Methods:**

Data were drawn from World Health Organization/United Nations Children’s Fund (WHO/UNICEF) estimates and the "Gavi, the Vaccine Alliance" administrative reports (1999–2018). Seven vaccines were assessed: third dose of polio vaccine (Pol3), first and second doses of measles‐containing vaccine (MCV1, MCV2), first and third doses of diphtheria‐tetanus‐pertussis vaccine (DTP1, DTP3), Bacillus Calmette–Guérin (BCG), and third dose of hepatitis B vaccine (HepB3). Linear spline regression, with knots in 2006 and 2018, was validated.

**Results:**

Between 1999 and 2023, coverage of all seven vaccines increased. WHO/UNICEF data showed Pol3 rising from 27% to 68%, MCV1 from 31% to 55%, DTP1 from 15.2% to 67%, DTP3 from 27% to 60%, and BCG from 38% to 68%, with MCV2 growing from 2% to 42% and HepB3 peaking at 67%. Spline regression revealed rapid growth from 1999 to 2006, slower increases from 2007 to 2018, and declines from 2019 to 2023. Gavi data mirrored these patterns, with DTP3 rising by 7.96% annually from 1999 to 2006 and DTP1 falling by 0.30% from 2007 to 2018. EPI centers expanded by 159.78 per year (2001–2006) and 74.12 (2007–2018).

**Conclusions:**

Afghanistan’s immunization coverage increased substantially until 2006, grew more slowly from 2007 to 2018, and declined after 2019. These patterns highlight the vulnerability of routine immunization programs to contextual challenges and suggest that sustaining coverage will require continued strengthening of routine services, monitoring subnational disparities, and implementing conflict-sensitive strategies.

## Introduction

Immunization is a significant public health intervention that saves millions of lives annually by controlling and even eliminating several infectious diseases [1]. Vaccination is essential not only for reducing disease mortality and morbidity but also for reducing economic burdens by preventing medical expenses and productivity losses [2]. In addition, vaccines have been linked to improving cognitive and educational outcomes, indicating that they can play a significant long-term role in both individual and societal well-being [2].

Global vaccine coverage increased significantly between 1980 and 2019, particularly in the 1980 s, and continued to rise in the following decades, albeit at a slower pace[3]. In the Middle East and North Africa, routine immunization coverage increased, particularly for the diphtheria, tetanus, and pertussis vaccine (DTP) vaccine [3]. After the COVID-19 pandemic, there were disparities in vaccine coverage, especially in low and lower-middle-income countries, where disruptions in immunization services exacerbated pre-existing inequalities and left millions of children unprotected [4].

Afghanistan has expanded its vaccination schedule from the original six vaccines since the start of its national immunization program in 1978, and it now protects children against ten childhood diseases [5]. However, in 2023, more than 460,000 children in Afghanistan did not receive any vaccines [6]. In that same year, DTP vaccination coverage in Afghanistan was 67%, compared with 85% for the Eastern Mediterranean Region (EMR) overall [7]. Although vaccine coverage has improved in Afghanistan, several challenges remain, including restricted access to healthcare facilities and inadequate health awareness [8].

Afghanistan’s under-five mortality rate remains among the highest globally, in part because of low vaccine coverage [9].

However, a knowledge gap remains regarding the long-term trends in childhood routine vaccination coverage in Afghanistan. Therefore, we aimed to provide an analysis of vaccination trends over the last two decades, along with descriptive, context-based insights to inform future public health interventions in Afghanistan.

## Methods

### Study design

This time-trend ecological analysis aimed to examine changes in routine childhood immunization coverage and the number of Expanded Programme on Immunization (EPI) centers at the national level in Afghanistan between 1999 and 2023. Seven key vaccines were analyzed: the third dose of polio vaccine (Pol3), the first and second doses of measles-containing vaccine (MCV1 and MCV2), the first and third doses of DTP (DTP1 and DTP3), the Bacillus Calmette–Guérin vaccine (BCG), and the third dose of hepatitis B vaccine (HepB3).

### Data sources

Data were extracted from two main sources, including WHO/UNICEF (WUENIC) for the period 1999–2023, as well as the "Gavi, the Vaccine Alliance" for the period 1999–2018. WUENIC data represent estimates of national immunization coverage, while Gavi data reflect administrative coverage figures reported by national authorities. Gavi, established in 2000, is a global public–private health partnership committed to improving access to immunization in low-income countries.

### Vaccine coverage definition

The WUENIC provided standardized definitions for various vaccine coverage metrics. Pol3 refers to the percentage of surviving infants who received a 3rd dose of polio-containing vaccine, which may be either oral or inactivated polio vaccine. MCV1 indicates the percentage of surviving infants who received the 1 st dose of measles-containing vaccine, while MCV2 specifies the percentage of children who received the 2nd dose of measles-containing vaccine according to the nationally recommended schedule. DTP1 and DTP3 denote the percentage of surviving infants who received the 1 st and 3rd doses, respectively, of diphtheria–tetanus toxoid–pertussis containing vaccines. BCG represents the percentage of live births that have received one dose of the BCG vaccine in a given year. HepB3 is the percentage of surviving infants who received the third dose of hepatitis B-containing vaccine following the birth dose.

Administrative coverage offers another method for calculating vaccine uptake based on doses administered. This metric is calculated as the number of doses administered through routine services divided by the number in the target group, multiplied by 100.

### Data collection and quality assurance

National immunization coverage data were obtained from the WUENIC estimates. In contrast, administrative immunization coverage data and the number of EPI centers were extracted from Gavi’s publicly accessible documentation. Countries supported by Gavi submit immunization-related documents, which Gavi compiles, manages, and makes accessible. All available and accessible documents were reviewed, comprising more than 60 items, including Afghanistan Annual Progress Reports, Joint Appraisal Reports of Afghanistan, Approved Proposals for Afghanistan, and Gavi Annual Progress Reports. These documents span the last two decades. From these, administrative immunization coverage data for 1999–2018 and EPI center data for 2001–2018 were extracted. Although immunization coverage data were available for 1999 and 2000, no data on EPI centers were found for these years. Notably, MCV2 and HepB3 were introduced into Afghanistan’s routine immunization schedule in 2004 and 2006, respectively; thus, data for these vaccines are available only from those years onward. To ensure data quality, reports with inconsistencies were excluded from the final data set.

### Statistical analysis

Descriptive trends in immunization coverage and the number of EPI centers were initially visualized using graphical charts. For each vaccine, coverage was reported at the start, end, and peak points of the study period. Changes in the number of EPI centers were also illustrated to highlight the development of infrastructure over time. To analyze trends in immunization coverage and EPI center expansion, we employed linear spline regression. This method allows flexible modeling of nonlinear trends over time. Given the significant national political, social, and health transitions, the years 2006 and 2018 were selected as knots in the model. Between 2001 and 2006, Afghanistan experienced rapid and relatively sustained improvements in security and development with international assistance, particularly in the areas of healthcare and childhood vaccination [10, 11]. However, in 2006, the Taliban intensified their attacks. From this year onward, security steadily deteriorated [12–14]. From 2006 to 2018, the growth trajectory slowed, and increasing insecurity hindered the provision of healthcare services and the maintenance of vaccination coverage. Nevertheless, the government remained intact [15, 16]. However, in 2018, the presidential elections, originally scheduled for that year, were postponed until 2019. Following their eventual conduct, a deep political crisis emerged nationwide, which persisted until 2020 [17, 18].

We conducted sensitivity analyses using alternative knot points (± 2 years) to confirm the model's robustness. Model fit was assessed using Akaike Information Criterion (AIC) and Bayesian Information Criterion (BIC); both supported 2006 and 2018 as optimal knot placements. Model assumptions were subsequently validated. All statistical analyses and trend visualizations were performed using Stata version 17, with Microsoft Excel used for descriptive charting. A 95% confidence interval (95% CI) was applied.

### Ethical considerations

As the study used aggregated data without individual-level information, formal ethical approval was not required. However, the study was conducted under an approved research protocol by the Ethics Committee of Kerman University of Medical Sciences (Ethics Code: IR.KMU.REC.1403.205).

## Results

**Fig. 1:**
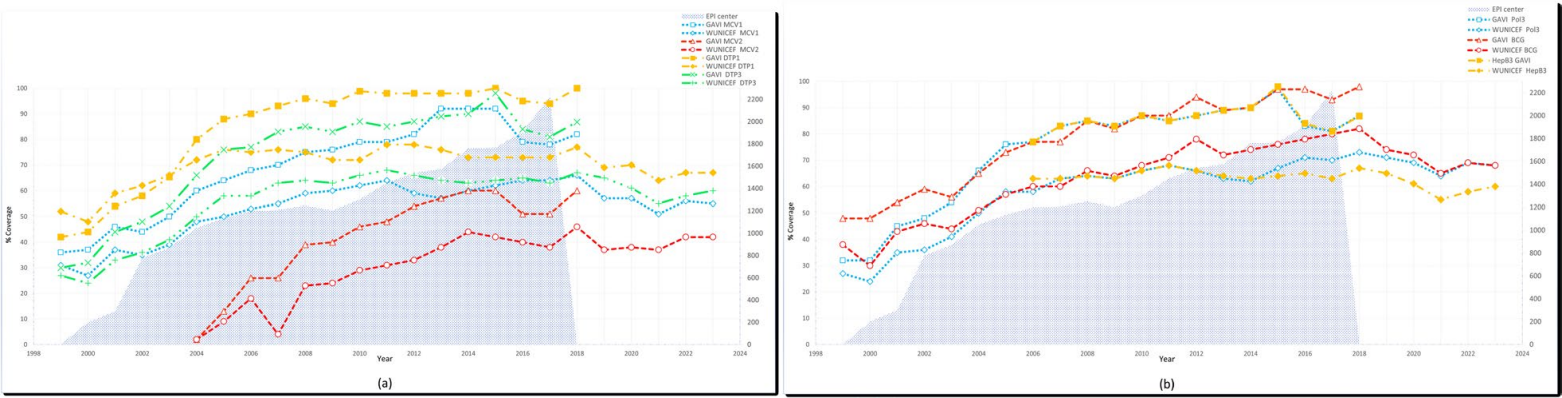
MCV–DTP (**a**) and Pol3–BCG–HepB3 (**b**) vaccination coverage and number of EPI Centers in Afghanistan, 1999–2023

The coverage of all seven vaccines has shown an increasing trend for most of the study period. According to the WUENIC data between 1999 and 2023, Pol3 rose from 27% to 68%, MCV1 from 31% to 55%, DTP1 from 15.2% to 67%, DTP3 from 27% to 60%, and BCG from 38% to 68%. The highest coverage levels for these vaccines were 73% for Pol3, 66% for MCV1, 78% for DTP1, 68% for DTP3, and 82% for BCG. MCV2 and HepB3, which were introduced into the national immunization schedule in 2004 and 2006, respectively, also showed changes: MCV2 increased from 2% to 42%, while HepB3 changed from 63 to 60%, peaking at 46% and 67%, respectively (Fig. 1).

Data from Gavi for 1999–2018 showed a similar upward trajectory. During this period, the highest coverage recorded was 97% for Pol3, 92% for MCV1, 60% for MCV2, 100% for DTP1, 98% for DTP3, 98% for BCG, and 98% for HepB3. Over the study period, coverage increased as follows: Pol3 from 32% to 87%, MCV1 from 36% to 82%, MCV2 from 2% to 60%, DTP1 from 42% to 100%, DTP3 from 30% to 86.8%, BCG from 48% to 98%, and HepB3 from 77% to 86.8%. Moreover, the number of EPI centers grew from 200 in 2001 to 2,225 in 2018 (Fig. 1).

**Fig. 2:**
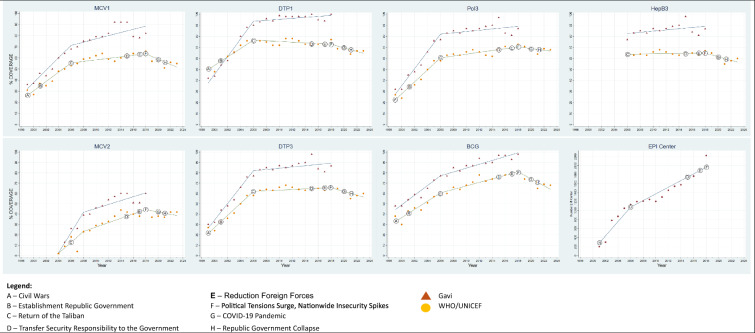
Trends in MCV1, MCV2, DTP1, DTP3, Pol3, BCG, HepB3 vaccination coverage and the number of EPI Centers in Afghanistan based on linear spline regression analysis of WHO/UNICEF estimates and Gavi administrative data, 1999–2023

Spline regression analysis identified three distinct periods with significant shifts in coverage trends: rapid growth (1999–2006), slower increases (2007–2018), and declines (2019–2023). According to the WUENIC data, the first period showed a statistically significant upward trend for all vaccines. In the second period, coverage continued to increase but at a slower pace. In the third period, coverage for nearly all vaccines declined. In the first period, DTP3 had the highest annual increase (slope = 5.81), while DTP1 had the lowest (slope = 2.82). MCV2 coverage, which began in 2004, also experienced rapid growth. In the second period, coverage continued to rise, though more slowly, with only DTP1 showing a decline. In the third period, BCG showed the most significant annual decline (slope = −3.37), while Pol3 experienced the smallest decline, which was not statistically significant (Table 1; Fig. 2).

**Table 1:** Trends of vaccination coverage in Afghanistan based on linear spline regression analysis of WHO/UNICEF estimates and Gavi administrative data, 1999–2023

Vaccine	Period 1 1999–2006	Period 2 2007–2018	Period 3 2019–2023	*Total Period
	Coefficient (95% CI)	Coefficient (95% CI)	Coefficient (95% CI)	Coefficient (95% CI)
WHO/UNICEF estimates				
BCG	4.00 (3.13, 4.86)	1.70 (1.26, 2.14)	− 3.37 (− 4.67, − 2.06)	1.57 (1.09, 2.05)
MCV1	4.19 (3.37, 5.01)	0.68 (0.27, 1.10)	− 2.40 (− 3.61, − 1.17)	1.06 (0.60, 1.51)
MCV2	**5.26 (2.45, 8.01)	**2.11 (1.20 3.02)	− 1.15 (− 3.27, 0.96)	1.91 (1.31, 2.51)
DTP1	2.82 (2.16, 3.49)	− 0.57 (− 1.12, − 0.02)	− 1.49 (− 2.87, − 0.11)	0.41 (− 0.004, 0.83)
DTP3	5.81 (5.09, 6.53)	0.30 (− 0.06, 0.66)	− 1.83 (− 2.91, − 0.75)	1.23 (0.67, 1.78)
Pol3	5.46 (4.75, 6.17)	0.83 (0.47, 1.19)	− 0.77 (− 1.84, 0.31)	1.63 (1.16, 2.10)
HepB3	–	0.08 (− 0.24, 0.40)	− 1.63 (− 2.50, − 0.76)	− 0.03 (− 0.57, − 0.03)
Gavi administrative report				
BCG	4.70 (3.88, 5.53)	1.86 (1.41, 2.30)	–	2.76 (2.35, 3.17)
MCV1	5.59 (4.14, 7.03)	1.43 (0.65, 2.21)	–	2.74 (2.10, 3.39)
MCV2	**9.78 (7.07,12.48)	**1.88 (0.92,2.84)	–	3.57 (2.47, 4.67)
DTP1	6.46 (5.77, 7.14)	− 0.30 (− 0.90, 0.31)	–	2.83 (1.95, 3.71)
DTP3	7.96 (6.88, 9.04)	0.60 (0.021,1.19)	–	2.93 (2.03, 3.82)
Pol3	7.80 (6.70, 8.89)	0.59 (0.002, 1.18)	–	2.87 (1.99, 3.75)
HepB3	–	0.55 (− 0.22, 1.32)	–	0.55 (− 0.22, 1.32)
Number of EPI centers	***159.78 (110.21,209.35)	74.12 (55.83, 92.41)	–	93.13 (78.45, 107.81)

^*^WHO/UNICEF Period 1999–2023 and Gavi Period 1999–2018

^**^MCV2 Period 1 2004–2008 and Period2 2008–2018

^***^Number EPI center Period 1 2001–2006

Gavi administrative coverage data showed a similar pattern to that of the WUENIC data, with a steep upward slope during the first period and slower increases in the second. In the first period, MCV2 rose sharply (slope = 9.78), and DTP3 increased by 7.96 annually. During the second period, coverage for all vaccines except DTP1 continued to increase; DTP1’s decline was not statistically significant (slope = − 0.30) (Table 1; Fig. 2).

The expansion of EPI centers followed an upward trend during the first and second periods. From 2001 to 2006, an average of 159.78 new centers were added each year (95% CI 110.21, 209.35). Between 2007 and 2018, the annual increase slowed to an average of 74.12 centers (95% CI 55.83, 92.41) (Table 1 and Fig. 2).

## Discussion

Our analysis of routine childhood immunization in Afghanistan from 1999 to 2023 showed that vaccination coverage and the expansion of health infrastructure were influenced by public health policy and political events. Using WUENIC and Gavi data, we found that coverage of all seven core vaccines increased substantially from 1999 to 2006, grew more slowly from 2007 to 2018, and declined from 2019 onward. Similarly, the number of EPI centers expanded rapidly until 2006 and then increased only modestly thereafter.

Measles-containing vaccine dose 2 (MCV2) coverage rose slowly after its 2004 introduction. This initial hesitancy likely reflects the vaccine’s novelty, caregivers’ unfamiliarity with an additional clinic visit, and the need to schedule a separate administration of the vaccine. By contrast, HepB3 achieved relatively high coverage from its 2006 inception, probably because it was co-formulated with DTP antigen and then administered as part of the pentavalent vaccine from 2010 onward.

Our spline analysis indicated that during 1999–2006, routine immunization coverage in Afghanistan rose most rapidly, with annual increases of up to 5.81 points for DTP3 and 5.46 points for Pol3. In this regard, Mashal and colleagues reported that between 2000 and 2003**,** national BCG coverage increased from 50.9% to 75.2% and DPT3 from 34.5% to 59.9% despite ongoing conflict [19]. Moreover, targeted mass campaigns early in this period demonstrated that even in conflict zones, high‐coverage thresholds were attainable. A measles drive conducted from December 2001 to May 2002 in five central provinces achieved over 90% coverage in two provinces and 91% among children aged 6–59 months in Kabul [20]. This suggests that the initial increase can be attributed to both the restoration of security corridors and the rapid scaling up of mobile teams, EPI staffing, international funding, and concerted efforts to rebuild primary and secondary healthcare [20, 21]. During the study period, Afghanistan received substantial external support. External Health Expenditure, including both direct foreign transfers and transfers distributed by the government from foreign origin, increased from less than USD 50 million in 2002 to approximately USD 700 million in 2022 [22]. The amount of official international aid received, according to OECD data, increased sharply from less than USD 150 million in 1999 to over USD 6.5 billion in 2012. It then gradually declined in subsequent years, reaching USD 3.05 billion in 2023. This influx of international funding coincided with a period of rapid expansion in both routine immunization coverage and the EPI infrastructure [23].

From 2007 to 2018, vaccine coverage continued to rise, albeit at a slower rate compared to the period from 1999 to 2006. This deceleration in routine coverage is consistent with several community‐based surveys conducted in this country. In this regard, Shenton et al. found that in 2015, only 40.6% of Afghan children aged 1–4 years were fully vaccinated, with 42.4% under-vaccinated and 17.0% non-vaccinated. This highlights that, despite continued growth, absolute coverage remained far below national targets [24]. Another study also reported a 38.8% fully immunized rate among 12–23 months in 2012, with particular limitations in the third dose of pentavalent vaccine (50.1%) and measles (64.8%), highlighting persistent gaps in completing multi-dose schedules [25]. Urban–rural disparities, maternal education, antenatal care visits, and institutional delivery have been proposed as predictors of full coverage, suggesting that sociodemographic barriers and health-seeking behaviors can affect uptake even where services are available [24, 25]. Moreover, Akseer et al. demonstrated that provinces experiencing higher‐intensity conflict achieved smaller annual improvements in DPT3 and measles vaccination compared to relatively secure regions, indicating that insecurity reduced service delivery and uptake [26]. After 2006, the political revolutions led to security issues and disrupted supply chains and outreach activities [14, 21]. While EPI center numbers continued to rise, the rate of growth in immunization coverage slowed. Spline coefficients for most vaccines were reduced by roughly half, and some, such as DTP1, even showed a non‐significant decline in slope. Political transitions in 2014–2017, determined by contested elections, the transfer of security responsibility to Afghan forces, and the gradual drawdown of foreign troops, further challenged service delivery [27]. Since the 1970 s, Afghanistan has been affected by recurrent episodes of international, internal, and non-state armed conflicts. As a result of ongoing armed conflicts, between 1999 and 2001, approximately 5,000 people were killed annually. Following the intervention of multinational forces in 2001, the Taliban regime collapsed, giving way to the establishment of a republic. Consequently, the intensity of conflict and insecurity declined, and between 2002 and 2005, the number of annual deaths dropped to nearly one-fifth of its previous levels. However, internal armed confrontations between the government of Afghanistan and opposing groups persisted and intensified once again from 2006 onward, deaths rose back to earlier levels, and by 2018–2019, Afghanistan had become the site of one of the deadliest conflicts worldwide, with annual death tolls peaking at approximately 27,000 to 30,000. The escalation of violence and insecurity disrupted public health programs, including vaccination efforts. In 2021, with the intensification of conflict and the withdrawal of foreign forces, the government collapsed, further exacerbating barriers to healthcare access and immunization services [28]. While these factors are widely recognized as important determinants of vaccination coverage [19, 26, 29–32], this study interprets them in the context of historical events, fluctuations in international aid, and prior research, rather than testing them quantitatively.

Between 2013 and 2018, the Sehatmandi project, a program in Afghanistan aimed at improving healthcare services using a performance-based financing model, sought to improve basic and hospital care through contracting with non-governmental organizations. However, insecurity and funding reductions constrained routine vaccination, particularly in rural and conflict-affected provinces [33].

Our spline regression analysis showed that, from 2019 to 2023, routine immunization coverage in Afghanistan decreased, with negative annual slopes for all investigated vaccines. This downturn coincided with the COVID-19 pandemic, during which a comparative analysis in Laghman province found a 21.4% drop in total childhood immunizations between April and July 2019 and the same period in 2020. Specifically, measles and OPV4 coverage fell by 28% and DPT3 coverage by 23%, underscoring how lockdowns and health system disruptions can precipitate sharp declines in vaccine uptake [34]. In 2019, Afghanistan entered a phase of political crisis as the delayed 2018 presidential election plunged the country into contestation that persisted into 2020. Attacks on health facilities and personnel, forced displacement, and denial of humanitarian access reduced routine service delivery and left children in conflict‐affected areas unvaccinated [35]. Even before the COVID‐19 pandemic, this period was accompanied by reduced international aid and state capacity. The COVID-19 pandemic in 2020 further depleted health resources, suspended outreach, and reduced caregivers’ access to clinics, exacerbating pre-existing declines in routine immunization [36].

This study has some limitations. First, as an ecological trend analysis, it is limited to the use of national-level aggregate data only, as subnational data were not available, and, therefore, does not account for subnational and district-level variations in immunization coverage and EPI infrastructure. Therefore, future studies can provide valid and reliable subnational data to evaluate the effects of different factors on vaccination in Afghanistan. Second, discrepancies between WUENIC and Gavi administrative reporting can be attributed to methodological variations, which may lead to reporting bias or misclassification of actual coverage levels. Third, our spline regression technique is sensitive to the position and quantities of knots. As a result, we conducted sensitivity analyses in 2006 and 2018 to validate our knot placement; however, other specifications might have slightly different inflection points. Fourth, we could not control for potential confounders, such as vaccine stockouts or sudden shocks, including mass displacement of the population.

## Conclusions

The two-decade trajectory of routine childhood immunization in Afghanistan highlights the importance of political stability, security, and sustained international support. The rapid increase from 1999 to 2006 demonstrates the transformative potential of coordinated mass campaigns, mobile outreach, and health-system rebuilding. However, the subsequent periods of insurgency resurgence, contested political transitions, and the COVID-19 pandemic reveal the vulnerability of these improvements. Future efforts should prioritize district-level surveillance to detect and address pockets of low coverage and integrate routine immunization. Qualitative and mixed-methods studies are necessary to identify barriers in insecure settings and assess the effectiveness of community-led interventions. Conflict-sensitive strategies and engagement with local leaders will be required to sustain immunization gains and protect Afghanistan’s children against vaccine-preventable diseases.

## Data Availability

The data that support the findings of this study are available from the corresponding author upon reasonable request.

## Declarations

### Declaration of use of AI

While preparing this work, the authors used ChatGPT 3.5 to correct the text and make it fluent. After using this tool/service, the authors reviewed and edited the content as needed and take full responsibility for the publication's content.
